# Kikuchi Disease With Marfanoid Features in a Background of Fever of Unknown Origin

**DOI:** 10.7759/cureus.104090

**Published:** 2026-02-22

**Authors:** Abdulaziz A AlTurki, Ibrahim Algosair, Saad M AlAlyani, Zahrah H Al-Faraj, Saud A BinNassar

**Affiliations:** 1 Internal Medicine, King Saud Medical City, Riyadh, SAU; 2 Anatomical Pathology, King Saud Medical City, Riyadh, SAU

**Keywords:** connective tissue disorder, fever of unknown origin, kikuchi-fujimoto disease, lymphadenopathy, marfanoid habitus

## Abstract

Kikuchi-Fujimoto disease (KFD) is a self‑limiting necrotizing lymphadenitis that usually affects young women. It can mimic infection, malignancy, or autoimmune disease, which often leads to diagnostic delays. Marfanoid habitus encompasses a constellation of physical traits associated with Marfan syndrome: tall stature, disproportionate limbs, arachnodactyly, joint hypermobility, and chest wall deformities. Co-existence of KFD and prominent marfanoid features has not previously been reported. Based on a structured PubMed/MEDLINE search from database inception through January 2026 using MeSH terms and keyword combinations for “Kikuchi disease,” “Marfan syndrome,” and “marfanoid habitus,” no prior cases describing this overlap were identified. This co-occurrence is clinically relevant because the presence of connective-tissue features may broaden the differential diagnosis, particularly toward autoimmune or heritable syndromes, thereby complicating the evaluation of fever of unknown origin. We describe a Middle Eastern adolescent who presented with fever of unknown origin and necrotic cervical lymphadenopathy. A remittent fever pattern was documented via a fever diary, and physical examination revealed numerous features of marfanoid habitus. Laboratory and imaging investigations excluded infectious, autoimmune, and malignant causes. Excisional lymph‑node biopsy confirmed necrotizing lymphadenitis consistent with KFD. Careful avoidance of empiric antimicrobial therapy in clinically stable patients undergoing evaluation for fever of unknown origin, particularly when atypical physical findings complicate the clinical picture.

## Introduction

Kikuchi-Fujimoto disease (KFD), or histiocytic necrotizing lymphadenitis, is a rare, benign inflammatory disorder characterized by fever and subacute necrotizing lymphadenopathy [1]. It was first described in Japan in 1972 and predominantly affects young adults, with a strong female preponderance [1]. Leucopenia and anemia are common laboratory findings, and the disorder is self‑limiting in most cases. Histologically, KFD presents as necrotizing lymphadenitis with focal areas of cell death containing abundant apoptotic debris and fragmented nuclear material. These regions are typically surrounded by collections of histiocytes and activated lymphocytes. A distinguishing feature is the absence of neutrophils and eosinophils within the necrotic areas. On immunohistochemical analysis, the inflammatory infiltrate is predominantly composed of CD8-positive T cells, and the associated histiocytes commonly express myeloperoxidase, lysozyme, CD68, and CD4. Although its etiology remains unclear, infection triggers autoimmune responses have been proposed. KFD often mimics infectious mononucleosis, tuberculosis, lymphoma, or systemic lupus erythematosus, necessitating tissue diagnosis. Treatment is usually supportive; corticosteroids may be considered for severe disease [2]. The presence of systemic connective-tissue features in a patient presenting with fever and lymphadenopathy can further complicate the diagnostic evaluation, requiring concurrent consideration of autoimmune and heritable connective-tissue disorders. 

Marfanoid habitus describes a constellation of skeletal and soft-tissue features seen in Marfan syndrome and related connective tissue phenotypes, without major cardiovascular or ocular features [3]. The diagnosis of Marfan syndrome is based on the revised Ghent criteria, which incorporate family history, aortic root dilation, ectopia lentis, pathogenic FBN1 mutations, and a systemic score of ≥7 points. In patients with a positive family history, the presence of key features may suffice for diagnosis, whereas in those without family history, specific combinations of aortic, ocular, genetic, and systemic findings are required to confirm the diagnosis [3,4].

To assess whether an association between Kikuchi disease and Marfan-spectrum phenotypes has been previously reported, a structured literature search was performed in PubMed/MEDLINE from database inception through January 20, 2026. The search strategy incorporated both MeSH terms (“Kikuchi Disease,” “Marfan Syndrome”) and keyword combinations (“Kikuchi-Fujimoto disease,” “histiocytic necrotizing lymphadenitis,” “marfanoid habitus,” “marfanoid features,” and “MASS syndrome”). No date or language restrictions were applied. Titles and abstracts of all retrieved records were screened, and reference lists of relevant articles were manually reviewed. No prior case reports or case series describing an overlap between KFD and Marfan-spectrum/marfanoid phenotypes were identified.

Here, we describe an adolescent male with persistent fever and necrotic lymphadenopathy confirmed to be KFD, who also exhibited multiple marfanoid features, highlighting the diagnostic challenges posed by this unusual combination and underscoring the importance of meticulous evaluation of fever of unknown origin (FUO).

## Case presentation

A 16‑year‑old Middle Eastern male patient presented with a 10‑day history of high‑grade fever and a painful left submental mass. He also reported recent decreased appetite and generalized fatigue; no other symptoms were noted. A course of amoxicillin-clavulanate prescribed by his general practitioner a week prior to presenting to our hospital had no effect. Given the persistence of high-grade fever, lack of response to antibiotics, and associated lymphadenopathy, he was admitted to our internal medicine service for further evaluation and management.

His past medical history was significant for a prolonged febrile illness three years prior, lasting four weeks and ultimately labeled as fever of unknown origin (FUO). At that time, he was admitted under the pediatric service at our hospital with a seven-day history of high-grade fever reaching 40°C with a tender cervical and submandibular lymphadenopathy. Clinical examination revealed bilateral cervical and submental tender lymphadenopathy without other associated symptoms. He was up to date with his immunizations and had normal growth and development for his age. During childhood, he experienced infrequent febrile episodes, approximately one to two per year, commonly associated with a sore throat. These were treated conservatively with symptomatic therapy and resolved spontaneously within about eight days. During his inpatient evaluation, he received empirical intravenous ceftriaxone and azithromycin, in addition to antipyretic therapy with paracetamol and ibuprofen. Cholecalciferol supplementation was started for vitamin D deficiency. Laboratory findings at initial presentation (Table 1) were notable for leukopenia with neutropenia and elevated inflammatory markers. Comprehensive infectious and autoimmune evaluations were negative.

**Table 1:** Laboratory investigations at initial presentation three years prior CBC: Complete blood count; ANC: Absolute neutrophil count; ESR: Erythrocyte sedimentation rate; CRP: C-reactive protein; LDH: Lactate dehydrogenase; ANA: Antinuclear antibody; Anti-dsDNA: Anti–double-stranded DNA antibody; C3/C4: Complement components 3 and 4; PPD: Purified protein derivative (tuberculin skin test); CMV: Cytomegalovirus; EBV: Epstein–Barr virus

Test	Result	Reference range
WBC	3.13 ×10⁹/L	4.5–13.5 ×10⁹/L
Absolute neutrophil count (ANC)	1.25 ×10⁹/L	1.5–8.0 ×10⁹/L
Hemoglobin	11.4 g/dL	11.0–15.5 g/dL
Platelets	213 ×10⁹/L	150–450 ×10⁹/L
Peripheral blood film	Absolute neutropenia; no blasts, no atypical lymphocytes, no hemolysis	—
ESR	110 mm/hr	0–15 mm/hr
C-reactive protein	26.4 mg/L	0–5 mg/L
LDH	272 U/L	140–280 U/L
Fibrinogen	5.48 g/L	2.0–4.0 g/L
D-dimer	1.73 mg/L	<0.5 mg/L
COVID-19	Negative	Negative
Blood culture	Negative	No growth
PPD skin test	Negative	<15 mm induration (low risk)
Malaria (3 thick films)	Negative	Negative
Brucella (IgG, IgM)	Negative	Negative
Parvovirus B19 PCR	Not detected	Not detected
CMV IgG/IgM	Negative	Negative
EBV IgM	Negative	Negative
Toxoplasma IgG	Negative	Negative
Salmonella	Negative	Negative
ASO titer	<200 IU/mL	<200 IU/mL
ANA (IFA)	<1:80 (Negative)	<1:80
Anti-dsDNA	<30 IU/mL (Negative)	<30 IU/mL
C3	1.87 g/L	0.9–1.8 g/L
C4	0.33 g/L	0.1–0.4 g/L
Vitamin D (25-OH)	8.5 ng/mL	30–100 ng/mL

**Figure 1:**
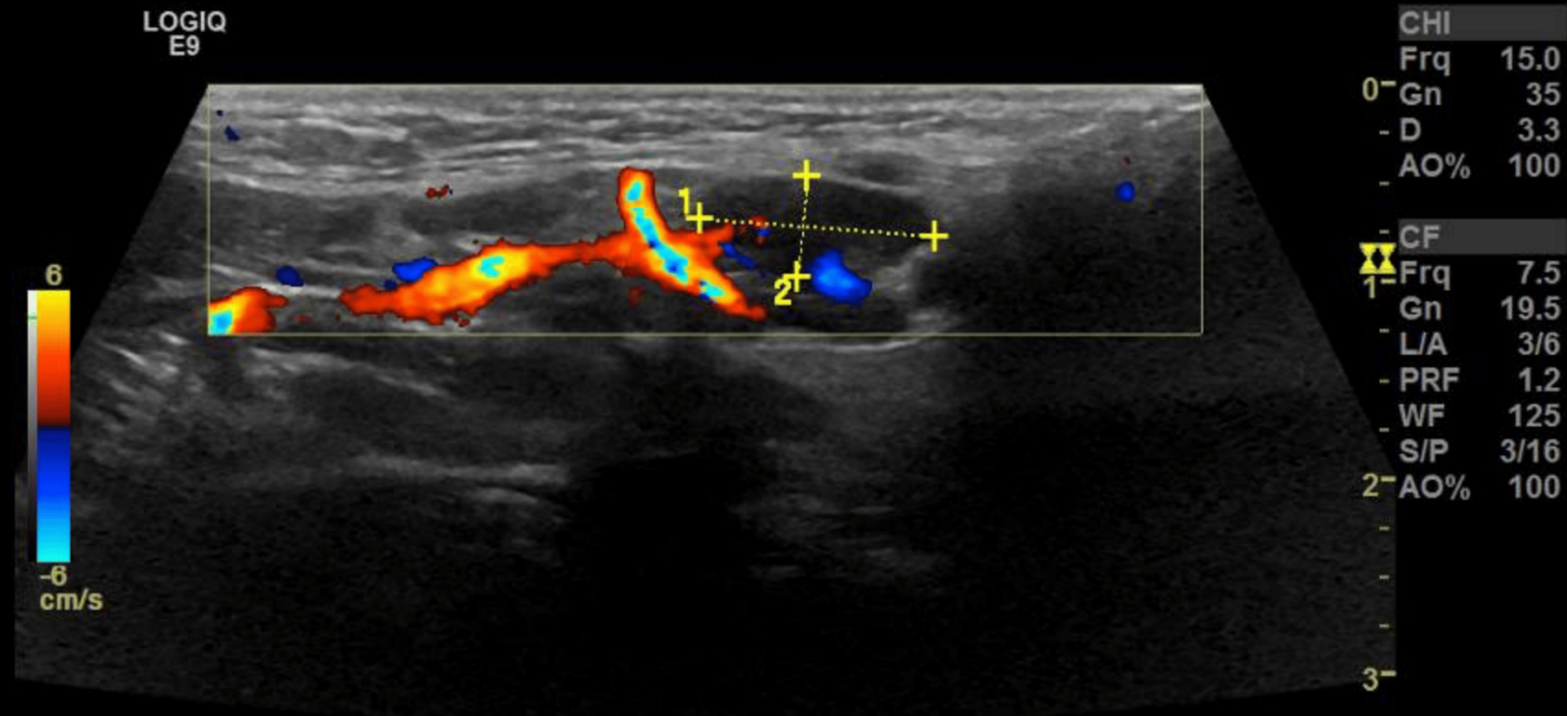
Ultrasound of the neck demonstrating a left submandibular lymph node measuring 1.2 × 0.51 cm Ultrasound image of the left submandibular region showing a well-defined lymph node measuring 1.2 × 0.51 cm in maximal dimensions. No sonographic features of abscess formation or extracapsular extension were identified.

Additionally, a transthoracic echocardiogram performed at that time revealed no abnormalities. To further evaluate the submandibular lymphadenopathy, a neck ultrasound was obtained, which demonstrated multiple submandibular lymph nodes. The largest measured 0.8 × 0.5 cm on the right side and 1.2 × 0.5 cm on the left side (Figure 1).

His febrile illness resolved within days of admission without a tissue diagnosis, and he was subsequently discharged home with outpatient follow-up. Over the following two years, he was monitored in the outpatient setting and remained asymptomatic, without recurrence of prolonged febrile episodes, lymphadenopathy, or new systemic symptoms. No key diagnostic investigation at the outpatient setting was done that changed his management. He maintained normal growth and daily functioning, demonstrated good academic performance, engaged appropriately in social activities, and reported a preserved appetite. His medical history was notable for pes planus managed with orthotics, bilateral myopia and strabismus for which corrective lenses were prescribed, as well as dental prognathism. There was no history of consanguinity and no family history of Marfan syndrome, marfanoid habitus, or autoimmune disease. He remained clinically stable until the current episode three years later.

**Figure 2:**
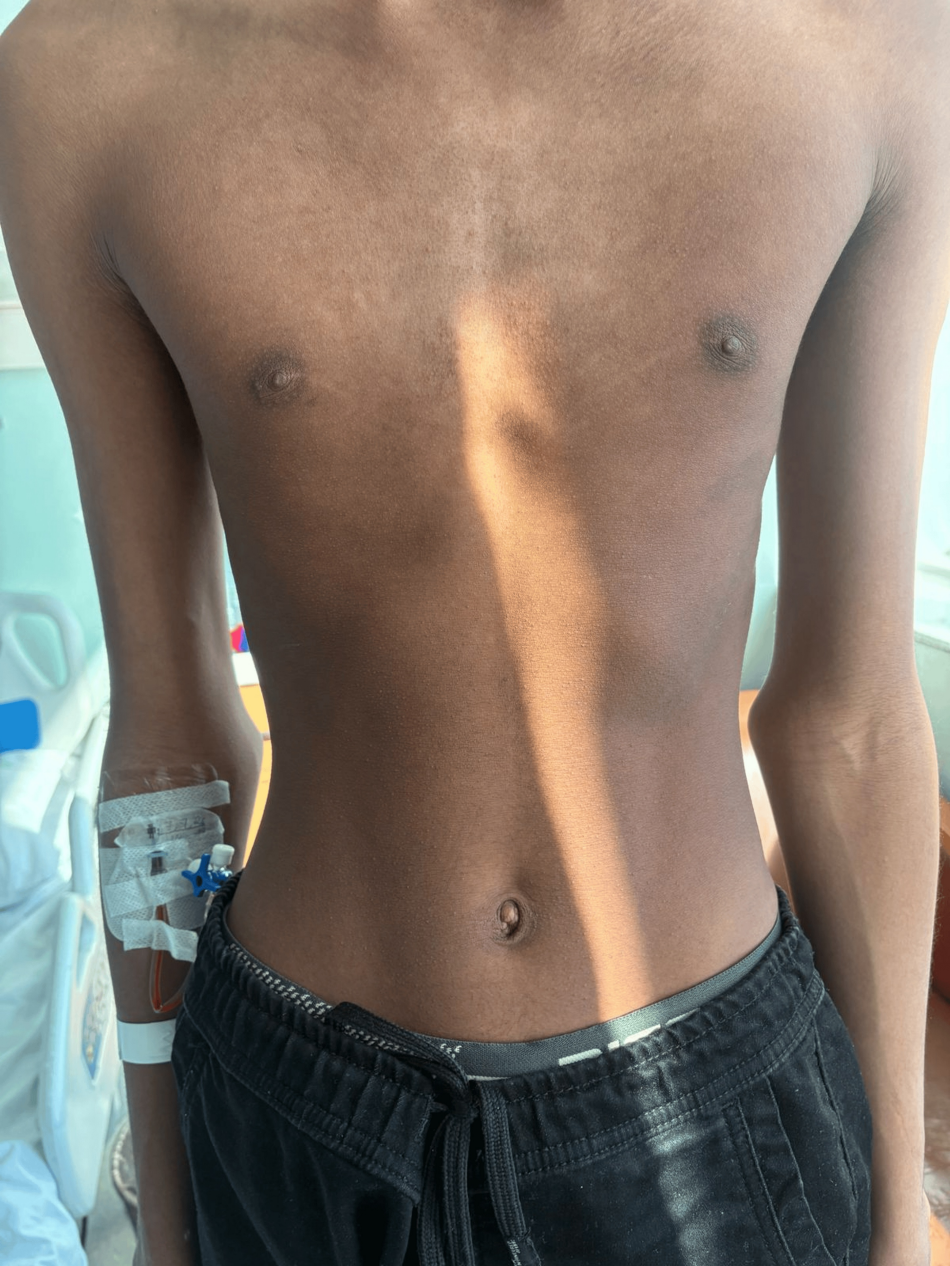
Pectus excavatum at the lower third of the sternum Clinical photograph illustrating the patient’s chest wall deformity (pectus excavatum), a component of his marfanoid habitus.

During his subsequent admission three years later, physical examination revealed a tall stature (179 cm) and marked underweight status (44 kg; body mass index 13.7 kg/m²). Both the patient and his family denied recent unintentional weight loss, reporting that his low weight was longstanding. Disproportionate limb length was evident, with an arm span of 191 cm. Vital signs showed a temperature of 38.4 C, pulse 113 beats/min, blood pressure 97/58 mmHg, respiratory rate 20 breaths/min, and oxygen saturation 97 %. Craniofacial features included prognathia and a high‑arched palate. Palpation revealed a tender, firm, slightly mobile 20 × 17 mm left submental lymph node, first the day after the onset of his fever. Chest inspection showed pectus excavatum (Figure 2). Cardiovascular and abdominal examinations were unremarkable.

**Figure 3:**
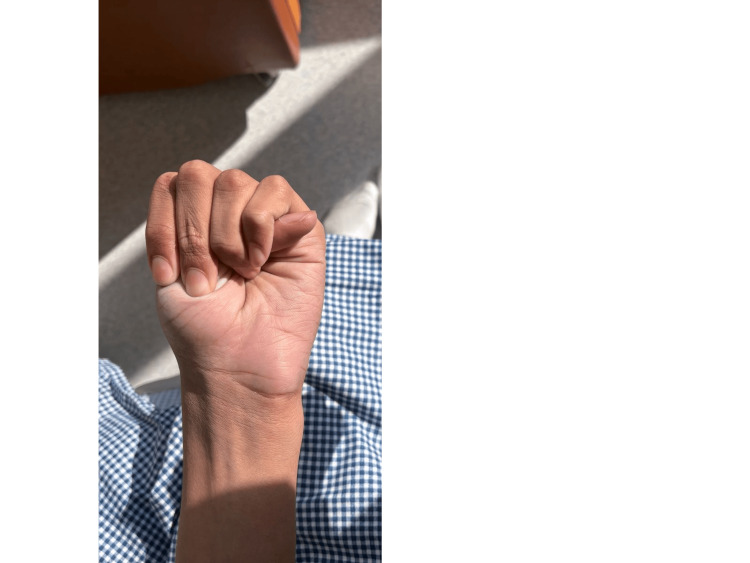
Positive Steinberg (thumb) sign The patient demonstrates the thumb sign, the thumb extends beyond the ulnar border of the hand when wrapped around the opposite wrist.

**Figure 4:**
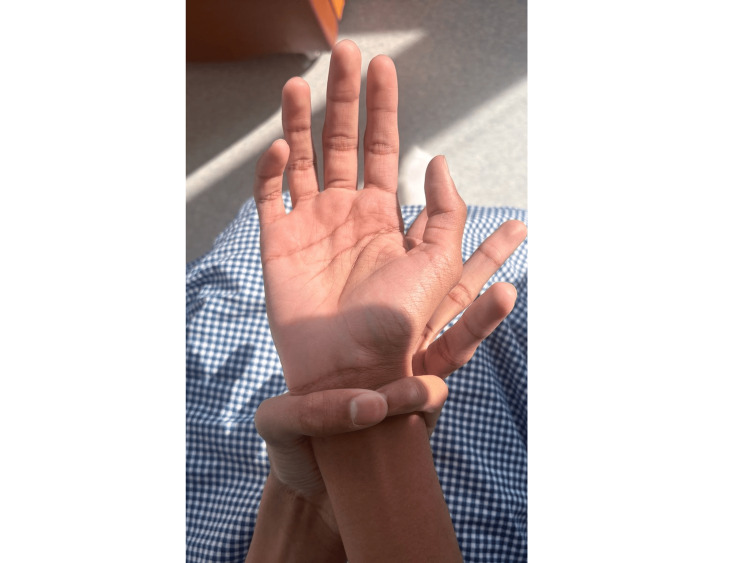
Positive Walker–Murdoch (wrist) sign The patient’s wrist sign shows the thumb and fifth finger overlapping when wrapped around the opposite wrist.

**Figure 5:**
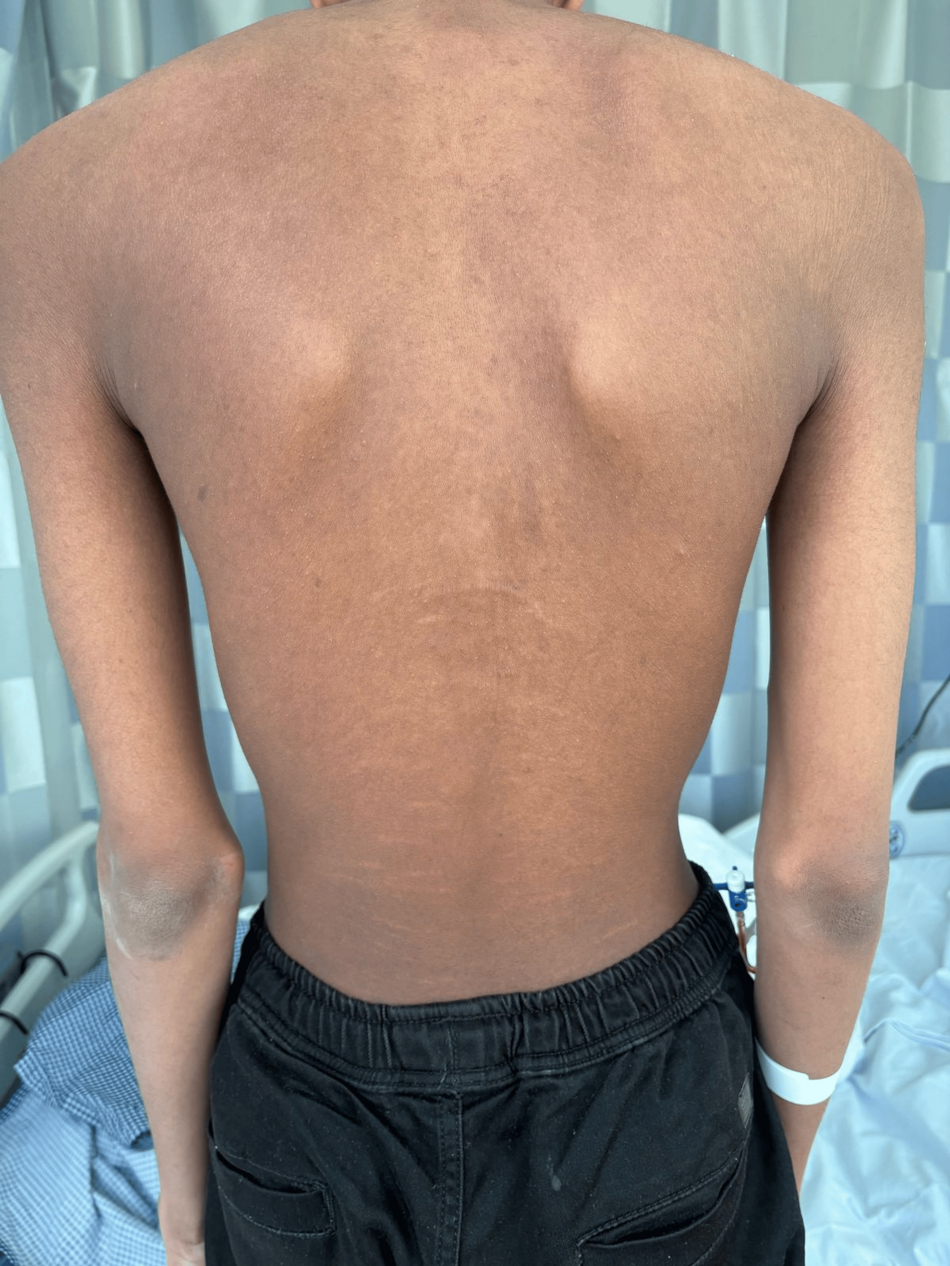
Bilateral lower‑back striae Photograph showing striae on the lower back, which can occur in connective‑tissue disorders and were part of the patient’s constellation of marfanoid features.

Musculoskeletal assessment demonstrated joint hypermobility, positive Steinberg and Walker-Murdoch signs, disproportionate body proportions (arm‑span‑to‑height ratio 1.13, upper‑ to lower‑segment ratio 0.88), bilateral pes planus, and lower‑back striae (Figures 3-5) show the positive Steinberg sign, the positive Walker-Murdoch sign. Skin examination revealed bilateral lower‑back striae; there was no rash or bruising. No other lymphadenopathy was noted. Ophthalmologic examination demonstrated myopia with strabismus but no evidence of ectopia lentis.

**Figure 6:**
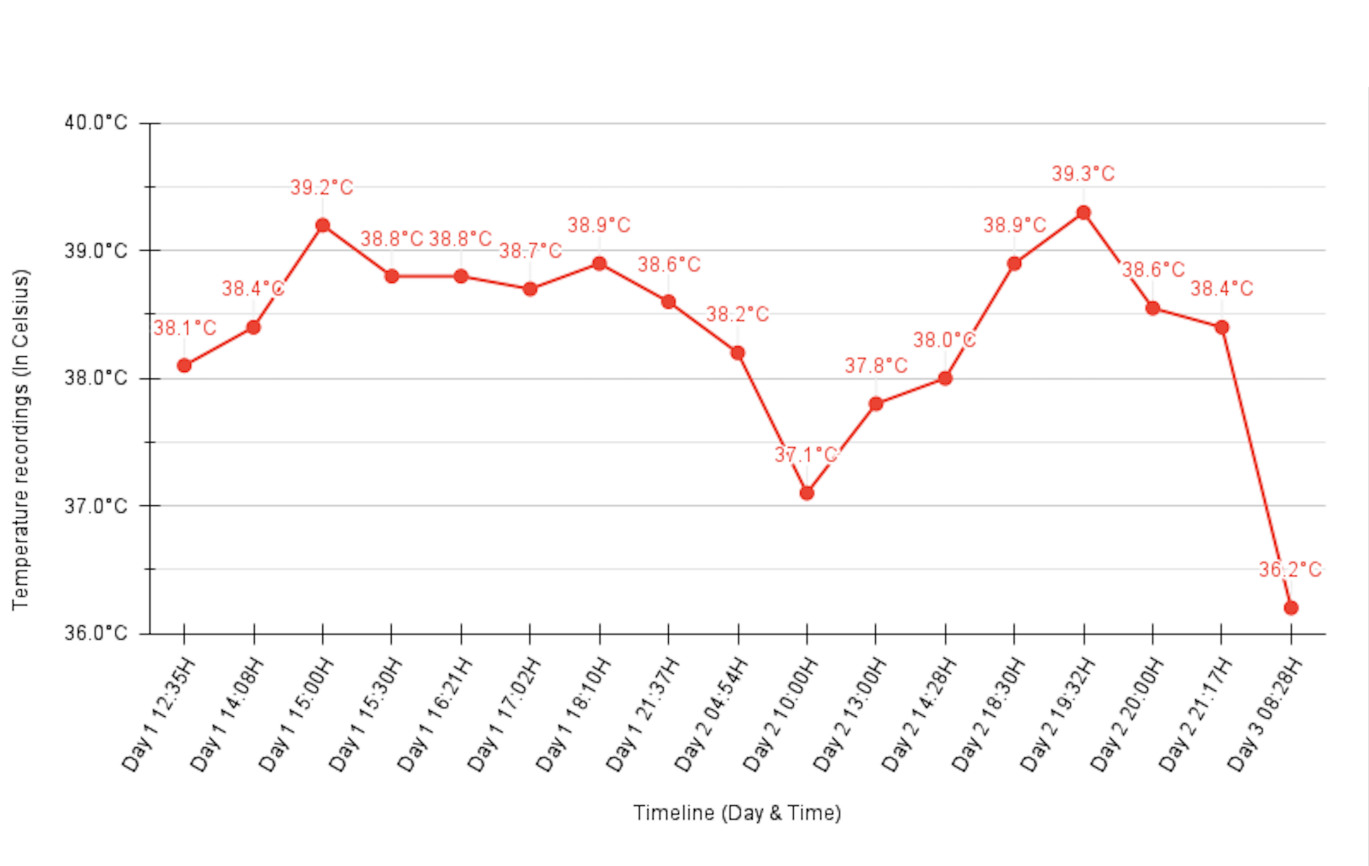
Remittent fever pattern documented in the patient’s diary The patient’s fever diary over the 10‑day admission. Note the remittent pattern, with two to three daily spikes up to 39‑40 °C and partial defervescence between peaks.

During hospitalization, we began prospectively charting his temperature using a fever diary for the following three days. The temperature was recorded with an oral thermometer, and the chart documented two to three daily spikes up to 39.2-39.3 °C with partial defervescence between peaks, consistent with a remittent pattern (Figure 6).

Laboratory tests (Table 2) demonstrated persistent leukopenia with relative lymphopenia and progressive neutropenia from a mild to moderate range; hemoglobin slightly trended downward, likely due to frequent sampling causing minor blood loss. Platelet counts were normal. Inflammatory markers were markedly elevated. Organ function, coagulation studies, and complements were normal. Potassium levels fluctuated within normal ranges while sodium levels were nearly normal; a slight variation in reported results was attributed to sampling or laboratory errors. Albumin, lactate dehydrogenase (LDH), and ferritin were within normal limits as well. Extensive microbiological and serologic evaluation was unrevealing. Autoimmune workup was unremarkable. HLA‑B27 and HLA‑DQ8 were negative. Molecular analysis of the FBN1 gene was negative for pathogenic or likely pathogenic variants.

**Table 2:** Selected laboratory trends during current hospitalization period BUN: Blood urea nitrogen; ESR: Erythrocyte sedimentation rate; CRP: C-reactive protein; ALT: Alanine aminotransferase; AST: Aspartate aminotransferase; LDH: Lactate dehydrogenase; ALP: Alkaline phosphatase

Parameter	Earliest value	Latest value	Reference range
Hemoglobin (g/dL)	13.75	12.9	13.5–18.0
Platelets (×10³/µL)	208.7	335	150–400
WBC (×10³/µL)	3.32	2.55	4.0–11.0
Neutrophil count (×10³/µL)	1.53	0.59	1.4–7.7
Lymphocytes count (×10³/µL)	1.35	1.63	1–5.5
Monocytes count (×10³/µL)	0.39	0.27	0.1-1.1
Eosinophils count (×10³/µL)	0.00	0.05	0.1-0.5
ESR (mm/hr)	60	76	0–30
CRP (mg/L)	43.7	46.2	<5
Creatinine (µmol/L)	63.9	53.8	63.6–110.5
BUN (mmol/L)	3.3	4.6	3-7.5
Sodium (mmol/L)	133	133	136–145
Potassium (mmol/L)	4.3	5.4	3.5–5.1
ALT (U/L)	9	14	0-55
AST (U/L)	25	23	8-48
Total bilirubin (µmol/L)	5.15	5.12	5-20
Direct bilirubin (µmol/L)	2.2	2.1	0-8
Alkaline phosphatase	88	67	55-160
Albumin (g/L)	41	41	32–46
LDH (U/L)	196	196	125–220
Ferritin (µg/L)	186.3	192.1	21.8–274.7

Transthoracic echocardiography demonstrated normal cardiac structure and function. The aortic root diameter at the sinus of Valsalva measured 2.6 cm (via the leading-edge method), corresponding to an estimated Z-score of approximately −1.6. The sinotubular junction measured 2.5 cm and the ascending aorta 2.4 cm. There was no evidence of mitral valve prolapse or other structural abnormalities.

**Figure 7:**
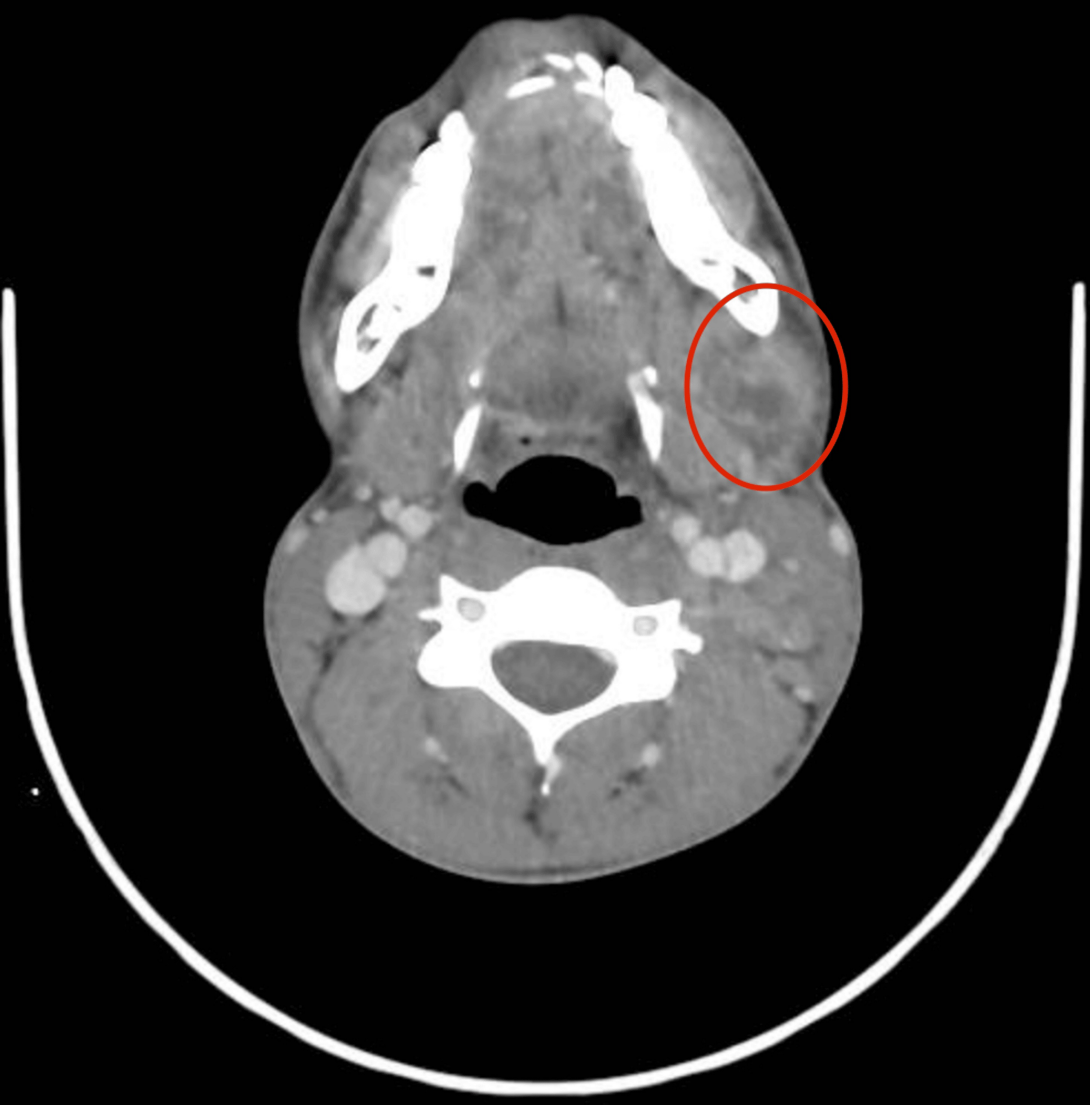
Contrast‑enhanced CT of the neck showing a necrotic lymph node. Axial CT image of the neck demonstrates a necrotic lymph node (20 × 17 mm) in the left submandibular region, circled in red.

Contrast‑enhanced computed tomography (CT) of the neck revealed multiple necrotic lymph nodes on the left side, with the largest measuring 20 × 17 mm. Figure 7 illustrates the contrast‑enhanced CT of the neck, demonstrating the necrotic lymph node.

**Figure 8:**
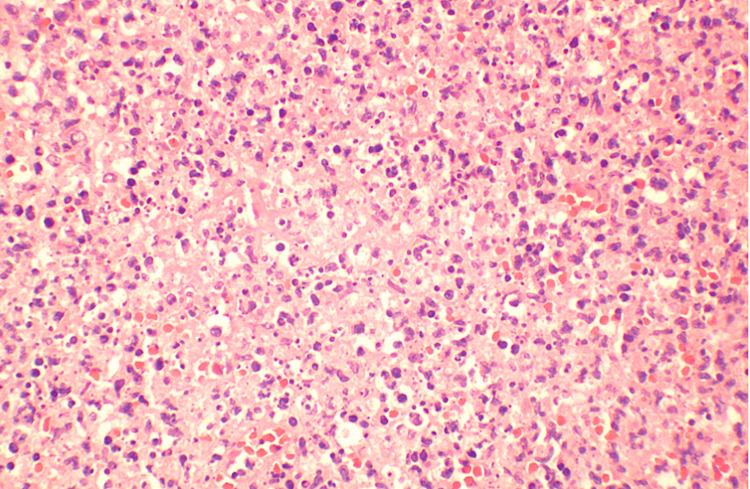
Hematoxylin and eosin (H&E) stain (40×) showing necrotic area and histiocytes High‑power H&E section of the excised lymph node reveals a pale necrotic area containing numerous histiocytes and karyorrhectic debris. Notably, neutrophils are absent.

**Figure 9:**
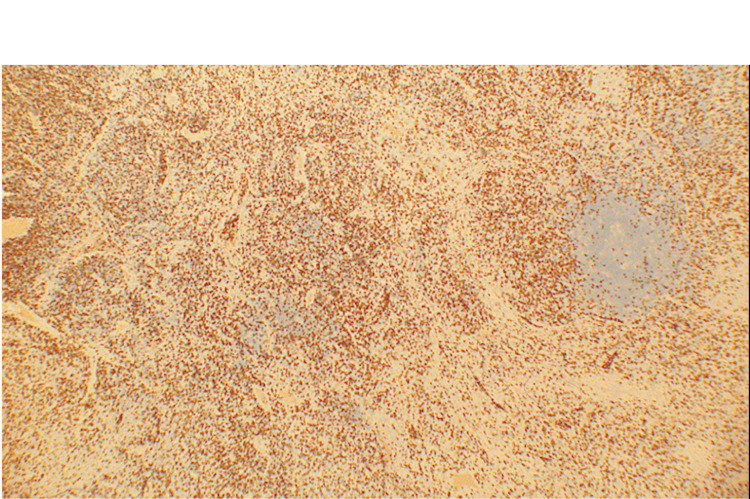
CD3 immunohistochemical stain highlighting T cells (10×) Immunohistochemical staining for CD3 demonstrates abundant T lymphocytes within the necrotic areas of the lymph node, supporting the diagnosis of necrotizing lymphadenitis.

**Figure 10:**
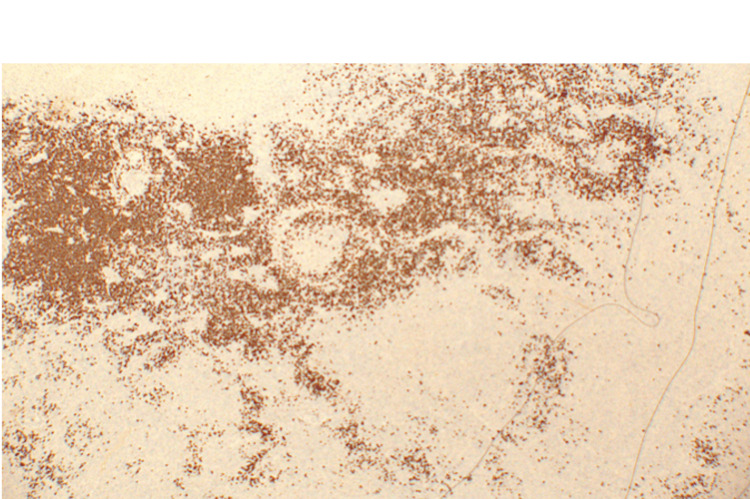
CD20 immunohistochemical stain highlighting B‑cell follicles (10×) CD20 staining accentuates residual reactive follicles with germinal centers, indicating preservation of some B‑cell architecture despite necrosis.

**Figure 11:**
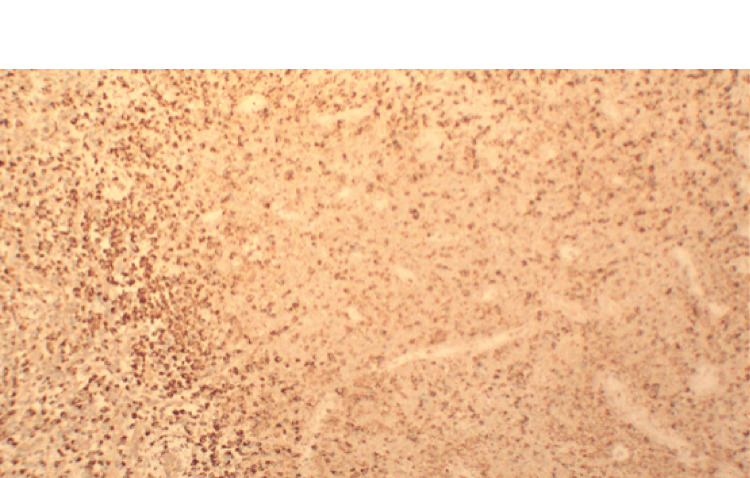
CD68 immunohistochemical stain highlighting histiocytes (10×) CD68 staining identifies numerous histiocytes surrounding the necrotic areas, a hallmark of Kikuchi–Fujimoto disease.

Given the persistence of fever and the presence of necrotic lymphadenopathy, a diagnostic excisional biopsy of the left submental lymph node was performed. Microscopic examination of the lymph node showed irregular paracortical areas of necrosis containing abundant histiocytes, eosinophilic granular material, and karyorrhectic debris. Residual reactive follicles were present, but intact neutrophils and epithelioid granulomas were absent. Immunohistochemical stains demonstrated abundant CD3‑positive T cells and CD68‑positive histiocytes around the necrotic areas, with CD20 highlighting residual B‑cell follicles and CD30 negative. Acid‑fast and fungal stains were negative. Figure 8 shows representative hematoxylin and eosin sections, while Figures 9-11 illustrate immunohistochemical staining highlighting CD3‑positive T cells, CD20‑positive B‑cell follicles, and CD68‑positive histiocytes, respectively. These findings confirmed necrotizing lymphadenitis consistent with KFD.

Management was conservative. Empiric antibiotics and corticosteroids were withheld, as the patient remained hemodynamically stable and not profoundly neutropenic. He was encouraged to maintain hydration and ambulate. Paracetamol was prescribed for temperatures exceeding 40 °C or upon request; however, no doses were administered during hospitalization. Serial complete blood counts and inflammatory markers were monitored. The day after the biopsy, his fever subsided. He was discharged with follow‑up appointments in general medicine, cardiology, and rheumatology. At three months, he remained afebrile with normal laboratory tests and stable cardiac assessments. Ongoing surveillance was arranged to monitor for recurrence of KFD and for development of connective‑tissue or autoimmune disorders.

## Discussion

This case illustrates an atypical presentation of Kikuchi-Fujimoto disease (KFD) in an adolescent male with prominent marfanoid features. KFD is a benign, self-limiting disease that most commonly affects young women and presents with fever and tender lymphadenopathy [1]. Pathogenesis remains incompletely understood; however, an immune-mediated mechanism has been proposed, potentially triggered by infectious agents, leading to a T-cell-driven response [5]. Although initially thought to be confined to Asia, KFD is now reported worldwide with regional variations; in Saudi Arabia and parts of the Middle East, KFD accounts for approximately 0.5-6.4% of all biopsy-confirmed cases of lymphadenopathy. A female predominance has been described, with a mean age of onset of 28 and 32 years [6,7], and recurrence remains rare (3-7%) [6]. Our patient deviated from the typical demographic profile; however, his presentation with fever, necrotizing lymphadenitis, leukopenia, and elevated inflammatory markers aligned with classical features of KFD [2]. Tissue histology confirmed the diagnosis and excluded tuberculosis (TB), lymphoma, or systemic lupus erythematosus (SLE), demonstrating hallmark features of abundant histiocytes and karyorrhectic debris, preserved reactive follicles, and absence of granulomas, neutrophilic or eosinophilic infiltration [2].

It is difficult to ascertain whether his initial presentation three years earlier represented a self-limited illness or an unrecognized episode of KFD. The reasons for not pursuing excisional biopsy during that admission remain unclear; however, the defervescence and regression of symptoms may have influenced that decision. KFD poses a diagnostic challenge, as it may be mistaken for more common infectious causes, particularly TB in endemic areas, or malignancy. Several reports describe patients receiving multiple courses of antibiotics or anti-tuberculous therapy before excisional biopsy ultimately established the diagnosis [5,8,9]. In both of his admissions, extensive infectious and autoimmune workups, including viral PCR testing, tuberculosis screening, zoonotic serologies, blood cultures, and autoimmune serologies, were unrevealing. His marfanoid habitus further complicated the diagnostic process, as lupus lymphadenitis, recognized as a manifestation within the spectrum of SLE and clinically overlapping with KFD, was considered unlikely in the setting of negative antinuclear titers, normal complement levels, and a self-limiting course, given that SLE typically follows a progressive multisystem course requiring sustained immunosuppressive therapy [2]. A malignant cause such as lymphoma would have persisted or progressed if left unaddressed. TB lymphadenitis was similarly unlikely in the presence of a negative Mantoux test on both occasions. In the absence of an excisional biopsy, however, a definitive diagnosis is difficult to establish, limiting certainty regarding the underlying etiology of his initial episode. Given that KFD is a recognized cause of fever of unknown origin (FUO), interpretation of his febrile pattern and clinical stability guided the diagnostic strategy.

FUO in many circumstances requires clinical expertise, comprehensive biochemical, and advanced imaging investigation. Empiric antimicrobials, corticosteroids, or anti-tuberculous treatment (in TB endemic areas) may be appropriate in clinically unstable patients, an organ-threatening disease process, or a profound neutropenia/immunocompromised state. Otherwise, indiscriminate therapy may delay definitive diagnosis and complicate interpretation of subsequent tests [10]. A prudent approach in such patients is warranted, starting with a thorough history and physical examination to synthesize a relevant list of differential diagnoses, with emphasis on more detailed illness characteristics, family, social, and travel histories, environmental exposures, and special habits to establish a relative framework of reasonable etiologies. To further aid in narrowing an exhaustive list of potential febrile diseases, a fever diary, though not a definitive diagnostic tool, carries value in aiding to identify a specific group of potential culprits [10]. Fever patterns are traditionally categorized as continuous, remittent, intermittent, or relapsing [11]. Intermittent fevers can be appreciated in TB, SLE, KFD, and lymphoma. Although the fever chart aided in narrowing the possible differential diagnosis, all of those diseases can cause night sweats, weight loss, and decreased appetite [6,10,11]. Correlating our patient's prior admission with the temporality of his symptomatology, and with his stable low BMI being unrelated to loss of appetite, suggested that his underlying etiology was benign and self-limiting in nature. Moreover, our patient remained hemodynamically stable, was only mildly neutropenic, and his initial imaging and laboratory evaluation were reassuring. This allowed for a timely excisional biopsy to reach a definitive diagnosis for the patient while avoiding unnecessary therapy exposure. The presence of systemic connective tissue features with fever and lymphadenopathy further complicated the diagnostic evaluation of this case, requiring simultaneous evaluation for autoimmune and heritable connective tissue conditions and careful evaluation of key disease features.

Diagnosis of Marfan syndrome is guided by applying the revised Ghent criteria. With a positive family history, the presence of ectopia lentis, aortic root dilation, or a systemic score of ≥ 7 points satisfies the diagnostic criteria. In the absence of a family history, the revised Ghent criteria require specific combinations of cardinal features; a diagnosis is confirmed when aortic root dilatation (calculated using the Z-score) is present in combination with ectopia lentis, a pathogenic FBN1 mutation, or a systemic score ≥7 points. Alternatively, in the absence of aortic root dilatation, the presence of ectopia lentis together with an FBN1 mutation previously associated with aortic disease is sufficient to establish the diagnosis [3,4,12]. Our patient demonstrated phenotypic features on physical examination, such as a tall, slender habitus, increased arm-span-to-height ratio, positive wrist and thumb signs, pes planus, and unexplained skin striae. Collectively, these findings raised suspicion for a Marfan-spectrum phenotype. Application of the revised Ghent criteria yielded a systemic score of 7 points. However, his transthoracic echocardiography, obtained to evaluate the aortic root and to exclude infective endocarditis as a potential contributor to his fever, revealed no pathology. The aortic root Z-score was approximately −1.6, well below the limit for dilatation, and there was no evidence of mitral valve prolapse or findings suggestive of infective endocarditis. A dedicated ophthalmologic evaluation did not reveal ectopia lentis. Genetic testing, including targeted analysis of FBN1, was performed and did not identify any clinically relevant variants. Therefore, despite matching skeletal features, the criteria for Marfan syndrome were not fulfilled. This framework is essential for distinguishing Marfan syndrome from related phenotypes along a clinical spectrum, such as Mitral valve prolapse, Aortic root dilatation (non-progressive), Skin striae, and Skeletal features (MASS) phenotype, and Isolated marfanoid habitus. Marfan syndrome carries a significant risk of progressive aortic dilatation and dissection, necessitating lifelong cardiovascular surveillance, while MASS phenotype includes skeletal and cutaneous features and may involve mitral valve prolapse but lacks progressive aortic enlargement [12]. Isolated marfanoid habitus describes individuals, as in this case, with marfanoid skeletal characteristics who do not meet systemic score requirements or major diagnostic criteria and therefore do not have a defined connective tissue disorder [4]. Although the risk of progressive aortic root dilation appears very low in individuals with isolated marfanoid features, periodic reassessment is reasonable due to diagnostic overlap within the Marfan spectrum [13].

To date, no definitive association has been demonstrated between isolated marfanoid habitus and autoimmune disease. To the best of our knowledge, there are no previously reported cases of individuals with marfanoid habitus developing KFD. More consistent relationships have been described between KFD and SLE via a possible underlying immunologic interplay, while only sporadic and rare links have been described between Marfan syndrome and autoimmune conditions [14-17]. Long‑term follow‑up is therefore essential, particularly given the potential association between KFD and systemic lupus erythematosus. Clinical evaluation with interval anti-nuclear antibody testing is warranted if new extra-nodal symptoms appear or with severe KFD episodes [14]. Repeat excisional biopsy should be considered when clinical and PET/CT findings are more suggestive of lymphoma than KFD (e.g., nodal conglomeration or necrosis, larger nodes, and higher FDG avidity) [15]. Clinicians should also remain vigilant for evolving cardiovascular manifestations in patients with marfanoid features; a serial echocardiogram every two to three years may be reasonable, particularly if associated with newly reported cardiovascular symptoms [13].

## Conclusions

Kikuchi-Fujimoto disease should remain a diagnostic consideration in adolescents who present with fever of unknown origin and necrotic cervical lymphadenopathy, even in populations where the disease is uncommon. A thorough evaluation to rule out infectious, autoimmune, and malignant causes is necessary, but tissue biopsy remains essential for definitive diagnosis. Adherence to fever‑of‑unknown‑origin guidelines, such as avoiding empiric antibiotics in clinically stable, immunocompetent patients, helps prevent delays in diagnosis and spares patients unnecessary treatments. Documenting fever patterns in a diary proved helpful in characterizing the remittent nature of the fever and guiding the timing of investigations. Although the presence of marfanoid features in this patient raised concern for an underlying connective‑tissue disorder, the absence of cardiovascular findings suggested marfanoid habitus rather than Marfan syndrome, underscoring the value of careful clinical assessment and appropriate follow‑up. Long‑term surveillance is warranted to monitor for recurrence of KFD and potential autoimmune or cardiovascular complications.
